# Survivin expression is associated with lens epithelial cell proliferation and fiber cell differentiation

**Published:** 2012-11-22

**Authors:** Miguel Jarrin, Fiona C. Mansergh, Michael E. Boulton, Lena Gunhaga, Michael A. Wride

**Affiliations:** 1 Visual Neuroscience and Molecular Biology Research Group, School of Optometry and Vision Sciences, Cardiff, University, Cardiff, Wales, UK; 2 Smurfit Institute of Genetics, School of Genetics and Microbiology, Trinity College Dublin, Dublin, Ireland; 3 Department of Anatomy and Cell Biology, University of Florida, Gainesville, FL; 4 Umeå Centre for Molecular Medicine (UCMM), By. 6M 4th floor, Umeå University, Umeå, Sweden; 5 Department of Zoology, School of Natural Sciences, Trinity College Dublin, Dublin, Ireland

## Abstract

**Purpose:**

Survivin (Birc5) is the smallest member of the inhibitor of apoptosis (IAP) protein family, which regulates the cell cycle/apoptosis balance. The purpose of this study was to examine Survivin expression in the embryonic chick lens, in chick lens epithelial cell cultures, and in the postnatal mouse lens.

**Methods:**

Survivin expression was examined using a combination of quantitative real-time polymerase chain reaction, western blotting, and immunocytochemistry. To correlate Survivin expression with the timing of proliferation, we determined the profile of cell proliferation in the developing lens using the cell cycle marker proliferating cell nuclear antigen (PCNA) in quantitative western blotting and immunocytochemistry studies. We also examined the expression of PCNA and the extent of denucleation using terminal deoxynucleotidyl transferase (TdT)-mediated biotin-dUTP nick-end labeling (TUNEL) of lentoids (lens fiber-like cells) during chick lens epithelial cell differentiation in vitro.

**Results:**

At embryonic day (ED) 4, Survivin immunostaining was present in two pools in lens epithelial cells and fiber cells: cytoplasmic and nuclear. The nuclear staining became more pronounced as the lens epithelial cells differentiated into lens fiber cells. At ED12, Survivin staining was observed in lens fiber cell nuclei containing marginalized chromatin, indicative of early denucleation events. Using western blotting, Survivin expression peaked at ED6, diminishing thereafter. This profile of expression correlated with the events in chick lens epithelial cell cultures: i) increased Survivin expression was associated with an increase in PCNA staining up to day 6 of culture and ii) downregulation of Survivin expression at day 8 of culture was coincident with a dramatic decrease in PCNA staining and an increase in TdT-mediated biotin-dUTP nick-end labeling in lentoids. In early postnatal mouse lenses, Survivin and PCNA were highly expressed and decreased thereafter during postnatal lens maturation.

**Conclusions:**

Survivin is expressed during chick and mouse lens development and in chick lens epithelial cell cultures. High levels of Survivin expression correlated with high rates of proliferation of lens epithelial cells at early stages of development. Downregulation of Survivin expression with development and its progressive localization to the nuclei of lens fiber cells was coincident with a decrease in cell proliferation and increased denucleation in differentiating lens fiber cells. These studies suggest an important role for Survivin as a dual regulator of lens epithelial cell proliferation and lens fiber cell differentiation.

## Introduction

Survivin (Birc5) is a member of the inhibitor of apoptosis protein (IAP) family originally discovered in the baculovirus [1]. Survivin is the smallest member of this family at 146 amino acids and 16.5 kDa. IAPs are characterized by one or more highly conserved baculoviral IAP repeat domains consisting of an approximate 70 amino acid, characteristic cysteine- and histidine-rich protein. Homologous IAPs have been found in nematodes, yeast, flies, and mammalian cells [1-3], and have roles as intrinsic regulators of the activity of initiator and effector caspases [4].

Structurally, Survivin is a unique IAP protein [5], organized as a stable dimer [6], containing only one baculoviral IAP repeat domain and a –COOH terminus coiled-coiled domain [7]. The special property of Survivin, which makes this protein different from the rest of the family, resides in its bifunctional role in controlling mitosis and inhibiting cell death. The tight regulation of cell division and cell death makes Survivin a master switch of organ and tissue homeostasis [8], an essential regulator of cell division [9], a modulator of microtubule dynamics and apoptotic and non-apoptotic cell death [10-12], and a stress response factor ensuring continued cell proliferation and survival [13].

Furthermore, alternative splicing of the Survivin transcript results in various isoforms that may have subtly different functions [14]. Additional studies regarding how Survivin expression is correlated to cell proliferation, apoptosis, and differentiation are required to better understand the role of Survivin in specific cell types, particularly during embryonic development.

Survivin is highly expressed in embryonic and fetal organs [15,16], but becomes restricted in its expression in adult tissues. Survivin knockout mice die at an early stage of development due to defects in mitosis [17]. Conditional deletion of Survivin neuronal precursor cells from ED10.5 resulted in apoptosis in these cells, resulting in death of the mutant mice shortly after birth [18]. Previous studies by our group have shown Survivin gene expression in the postnatal mouse lens [19,20] and downregulation of Survivin expression during cataract progression in the Sparc knockout mouse model [19]. This difference in Survivin gene expression between normal and cataractous lenses suggests that Survivin is a candidate factor for regulating the normal development and physiology of the vertebrate lens. The development of the lens depends on precise spatiotemporal control of lens epithelial cell proliferation and differentiation into lens fiber cells [21-23]. The differential regulation of cell proliferation in the lens is established as early as lens placode invagination in which the central part of the lens placode undergoes a reduction in the cell proliferation rate, while the peripheral part of the placode retains a high frequency of proliferation [24,25].

As development proceeds, proliferation becomes less frequent, and finally ceases completely in the primary and secondary lens fiber cells (LFCs [25]). At later stages of development, proliferating cells become localized to the outer parts of the peripheral lens epithelium, while proliferation is dramatically reduced in the central epithelial cells until the central cells become quiescent. Altogether, the structure of the lens is a consequence of a unique situation in which cells in different states (quiescence, proliferation, and differentiation) are located in specific compartments of the lens [22].

In addition, the differentiation of lens epithelial cells (LECs) into fiber cells is characterized by organelle loss, including denucleation [26]. This process may represent an “attenuated” form of apoptosis [27] in which the nuclear events are dissociated from the cytoplasmic and cell membrane processes that characterize classical apoptosis. Survivin could also be one of the factors involved in regulating organelle loss and denucleation during fiber cell differentiation, but the mechanisms that prevent full classical apoptosis during this process. In this study, we have analyzed the expression of Survivin in the developing lens in relation to cell proliferation and differentiation. Briefly, our results suggest that high expression of Survivin correlates with high rates of cell proliferation, whereas downregulation of Survivin expression is coincident with a decrease in lens epithelial cell proliferation and an increase in fiber cell denucleation.

## Methods

### Experimental animals

White Leghorn hens’ eggs (Henry Stewart, Co. Ltd, Louth, UK) were used to collect lenses from chick embryos between embryonic day (ED) 4 (Hamburger and Hamilton [HH] stage 24) and ED20 (HH stage 46 [28]) every two days. Lenses were also collected from mice at newborn (NB), P7, P14, and 4 weeks. The experiments were performed in accordance with UK legislation (Animals, Scientific Procedures, Act 1986) and the European directive (86/609/EEC) and conform to the ARVO statement for the Use of Animals in Ophthalmic and Vision Research.

### Tissue processing

Eyes were removed from chicken embryos and postnatal mice, washed in ice-cold phosphate buffered saline (PBS; 137 mM NaCl, 10 mM Phosphate, 2.7 mM KCl, pH 7.4), fixed for 24 h at 4 °C in 4% paraformaldehyde (PFA), washed with PBS and dehydrated through a graded series of ethanol and cleared in 50:50 ethanol: xylene for 30 min and then 100% xylene for 3 min. Tissues were subsequently infiltrated with paraffin wax (Fisher, Loughborough, UK), embedded, and sectioned at 7 microns.

### Immunostaining

Polyclonal rabbit anti-Survivin (FL142, Santa Cruz Biotechnology, Inc., Heidelberg, Germany) was diluted to 1:400 and incubated with sections overnight at 4 °C. Monoclonal antiproliferating cell nuclear antigen (PCNA, PC10; Abcam, Cambridge, UK) was diluted to 1:500 and incubated with sections for 1 h at room temperature. Antigen retrieval was performed using a citrate buffer (Vector Labs, Peterborough, UK). Anti-rabbit biotinylated antibody (Vector Labs) at 1:500 dilution was incubated with tissue sections for 1 h, washed three times, and then the immunoperoxidase ABC system (Vector Labs) was used with 3,3′-Diaminobenzidine (DAB; Vector Labs) as chromogen. For PCNA, a secondary fluorescent dye-coupled anti-mouse antibody, Alexa Fluor 488 (Invitrogen, Paisley, UK) was used at 1:500. As negative controls, omission of the secondary antibody and replacing the primary with mouse immunoglobulins were used.

### Chick lens epithelial cell cultures

Chick lens epithelial cell cultures were performed as described previously [29-31]. Briefly, lenses were removed at ED10, pooled, and placed in Tyrode’s saline containing gentamycin (50 μg/ml). Lens cells were dissociated from each other in 2.5% trypsin solution (Sigma Aldrich, Gillingham, UK) at 37 °C for 15 min using a 22 G needle and then centrifuged at 10,000 g for 2 min. The cell pellet was resuspended in 300 μl of medium 199 (Invitrogen, Paisley, UK) containing 10% fetal calf serum (Gibco) and gentamycin (Gibco), and then the cell suspension was filtered using a 40 μm falcon cell strainer (BD Biosciences, Oxford, UK). Twenty-four well plates were coated with 1.2 mg/ml of Matrigel (Invitrogen) and allowed to air dry. The wells were washed with medium 199 before 5×10^5^ cells/well were seeded. Incubation of cells was performed in a humid atmosphere at 37 °C in 5% CO_2_. Cells were allowed to attach and begin to spread for 24 h, and this was designated day 0 of culture. The medium was subsequently changed every day.

### Western blot

Protein was isolated from pooled embryonic chick lenses or postnatal mouse lenses using RIPA buffer (Upstate [Merck Millipore], Darmstadt, Germany) containing protease inhibitor cocktail (Sigma). Protein was collected from lens epithelial cell cultures at days 0, 2, 4, 6, and 8 (D0–8). Samples were incubated at 4 °C on a rotator for 30 min and then centrifuged at 13,000 × *g* for 30 min at 4 °C. The supernatant was removed, aliquoted, and stored at –20 °C. Protein concentration was determined using the Bio-Rad Protein Assay (Bio-Rad, Hemel Hempstead, UK), and 10 μg of total protein was loaded into thethe gel system using Laemmli buffer (Bio-Rad). Proteins were separated by 12% sodium dodecyl sulfate PAGE (SDS–PAGE) and transferred to a 0.2 μm nitrocellulose membrane (GE Healthcare Life Sciences, Little Chalfont, UK). The membranes were blocked for 1 h with 5% nonfat milk (Sigma Aldrich, Gillingham, UK) and incubated with polyclonal rabbit anti-Survivin (1:1000 dilution; Santa Cruz Biotechnology, Inc., Heidelberg, Germany), monoclonal anti-PCNA (1:5000 dilution; Sigma), or monoclonal anti-β-actin (1:10,000; Santa Cruz) primary antibodies and then goat anti-rabbit or anti-mouse HRP secondary antibodies (1:7500 dilution; Santa Cruz Biotechnology). ED6 (HH stage 29) chicken brain was used as positive control. Western blotting (WB) band intensity values were obtained using Labworks (Media Cybernetics, Rockville, MD), and β-actin was used as a housekeeping protein to normalize band intensity. The mean band intensities (normalized) for each protein at each time point were calculated along with the standard error, and for WB repetitions (n=3), ED12 was used as the calibrator to which all other band intensities for the various different samples were compared.

### TdT-mediated biotin-dUTP nick-end labeling

Labeling of fragmented DNA in lens sections or in fixed chick lens epithelial cell cultures was performed using the DeadEnd fluorometric TdT-mediated biotin-dUTP nick-end labeling (TUNEL) kit (Promega, Southampton, UK) following the manufacturer’s instructions. As positive control, sections were incubated 30 min with DNase II (5 U/μl; Roche, Welwyn Garden City, UK) before TUNEL labeling.

### Collection and preparation of lenses for RNA isolation and integrity

Chick or mouse lenses were immediately homogenized in TRIzol reagent (Invitrogen) using a tissue grinder (Wheaton, City, Country) and RNA isolated. For RNA isolation from chick epithelial dissociated primary cell cultures, the RNeasy Micro kit (Qiagen, Crawley, UK) was used. RNA was quantified using a spectrophotometer (GeneQuant II, GE Healthcare Life Sciences) at 260 nm and checked for RNA integrity via agarose gel electrophoresis.

### Quantitative real-time polymerase chain reaction

The primers for quantitative real-time polymerase chain reaction (QPCR) were QuantiTect Primer Assays (Qiagen). These primer sets for Survivin, glyceraldehyde 3-phosphate dehydrogenase and β-actin, are prevalidated, custom-designed, and proprietary (sequences were not made available). Survivin expression levels were examined with quantitative real-time PCR with SYBR Green Master Mix (Sigma) using a Roto-Gene 6000 (Corbett Research, Cambridge, UK). The data from QPCR were analyzed using the 2-ΔΔCt method [32], and melting curve analysis was performed to confirm primer specificity. QPCR was performed as follows: 95 °C for 5 min, then 40 cycles of 94 °C for 30 s, 60 °C for 30 s, and 72 °C for 30 s. Three different sample pools were used for each stage, and each PCR reaction was performed in duplicate. The average result was used for calculations. Results from each sample were calibrated using ED12 (HH stage 38).

### Statistical analysis

Data were analyzed using the SPSS 12 software package for Windows (IBM, Portsmouth, UK). Comparison between samples was performed using parametric tests: ANOVA (ANOVA), followed by the Tukey or Dunnett T3 post-hoc tests, was used to determine the degree of statistical significance, which was taken as significant if p<0.05.

## Results

### Survivin gene and protein and proliferating cell nuclear antigen protein expression in the chicken embryo lens

**Figure 1:**
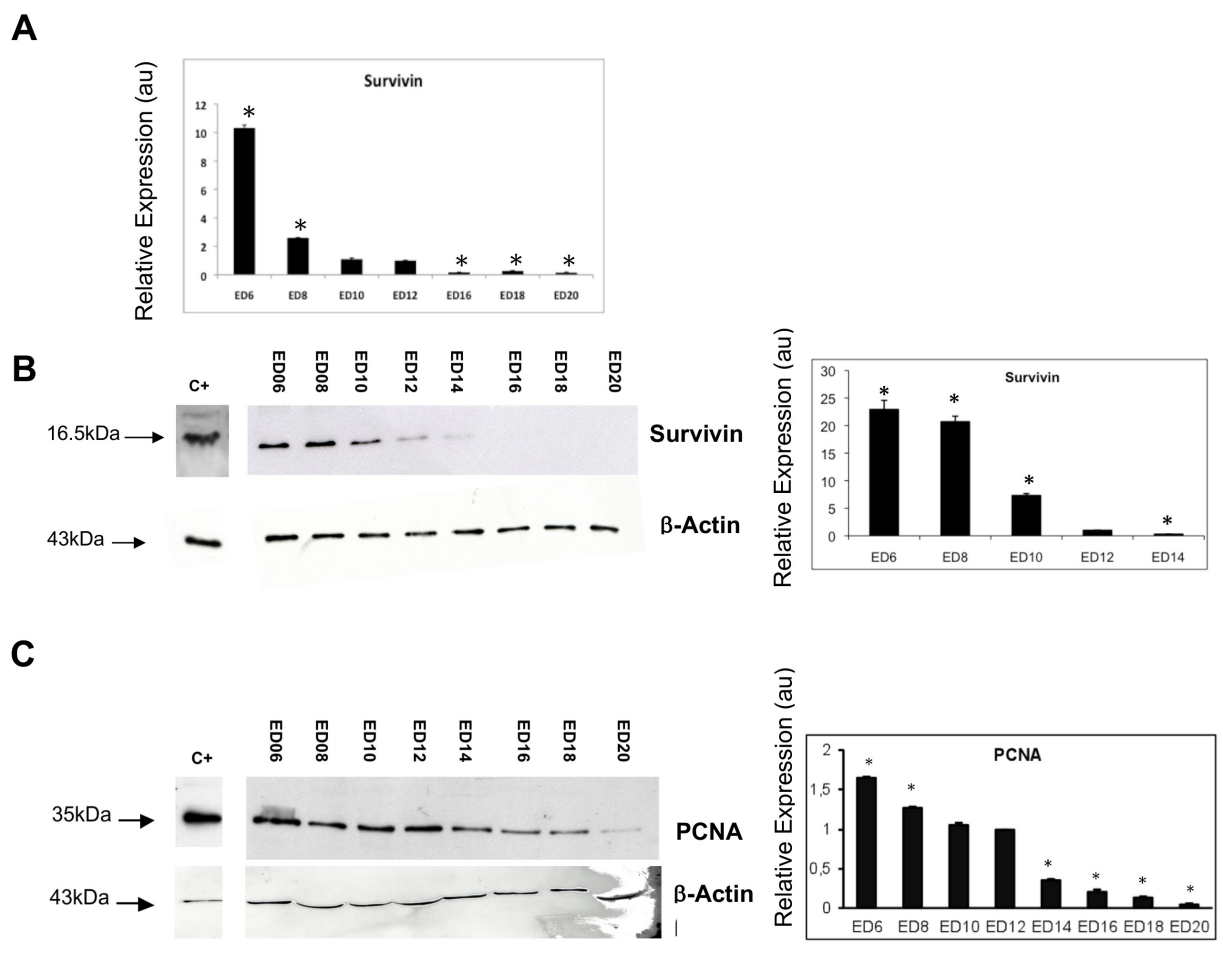
Survivin gene and protein and proliferating cell nuclear antigen (PCNA) protein expression in the chicken embryo lens. **A**: QPCR for Survivin mRNA expression during lens development revealed that Survivin mRNA was maximal at ED6 followed by dramatic downregulation at ED8 onwards. All stages except ED10 were statistically significantly different regarding the calibrator (ED12) to which all samples were normalized (*=p<0.05). **B**-**C**: Survivin and PCNA protein expression in the lens were analyzed between ED6 and ED20 (representative western blots are shown). ED12 was used as the calibrator to which all other stages were compared. **B**: There was a statistically significant decrease in Survivin expression from ED8 onwards, and all stages were significantly different regarding the calibrator (ED12; *=p<0.05; n=3) **C**: PCNA (35 kDa) expression was detected at all stages, but was downregulated as lens development proceeded. With the exception of ED10, statistically significant differences were found between the expression levels of PCNA at all stages compared to the calibrator ED12 (*=p<0.05; n=3). Chicken embryo brain (ED6) was used as positive control C+ in each case. Protein expression was quantified with densitometry using the scan program and normalized with respect to β-actin protein (n = 3) as a housekeeping control. Error bars represent the standard error of the mean. au=arbitrary units for relative levels of expression.

Survivin expression in the developing chick lens was confirmed using QPCR (Figure 1A). Survivin mRNA was maximal at ED6 followed by dramatic downregulation at ED8 onwards. All stages except ED10 were statistically significantly different regarding the calibrator (ED12), to which all samples were normalized (*=p<0.05).

To examine Survivin expression in the developing chick lens in relation to changes in lens cell proliferation, we then studied the expression of Survivin and PCNA protein expression using WB (Figure 1B,C). The Survivin 16.5 kDa wild-type band was detected at all stages of development examined up to and including ED14, but was not detected from ED16 onwards (Figure 1B). There was a statistically significant decrease in Survivin expression from ED8 onwards, and all stages were significantly different regarding the calibrator ED12 (*=p<0.05; n=3). From ED14, the levels of Survivin expression were low to negligible in WB (Figure 1B). To assess overall cell proliferation changes in the developing chick lens, WB was performed for PCNA (Figure 1C). WB revealed intense expression of the PCNA 35 kDa band during the earliest stages of chick lens development, which diminished as development proceeded. Relative levels of expression, determined with densitometry analysis and presented graphically, revealed statistically significant decreases in PCNA expression with development (Figure 1C; p<0.05), except between ED10 and the calibrator ED12. Thus, downregulation of Survivin expression accompanied a decrease in cell proliferation in the developing chick lens.

### Spatiotemporal localization of Survivin in the chick embryo lens

**Figure 2:**
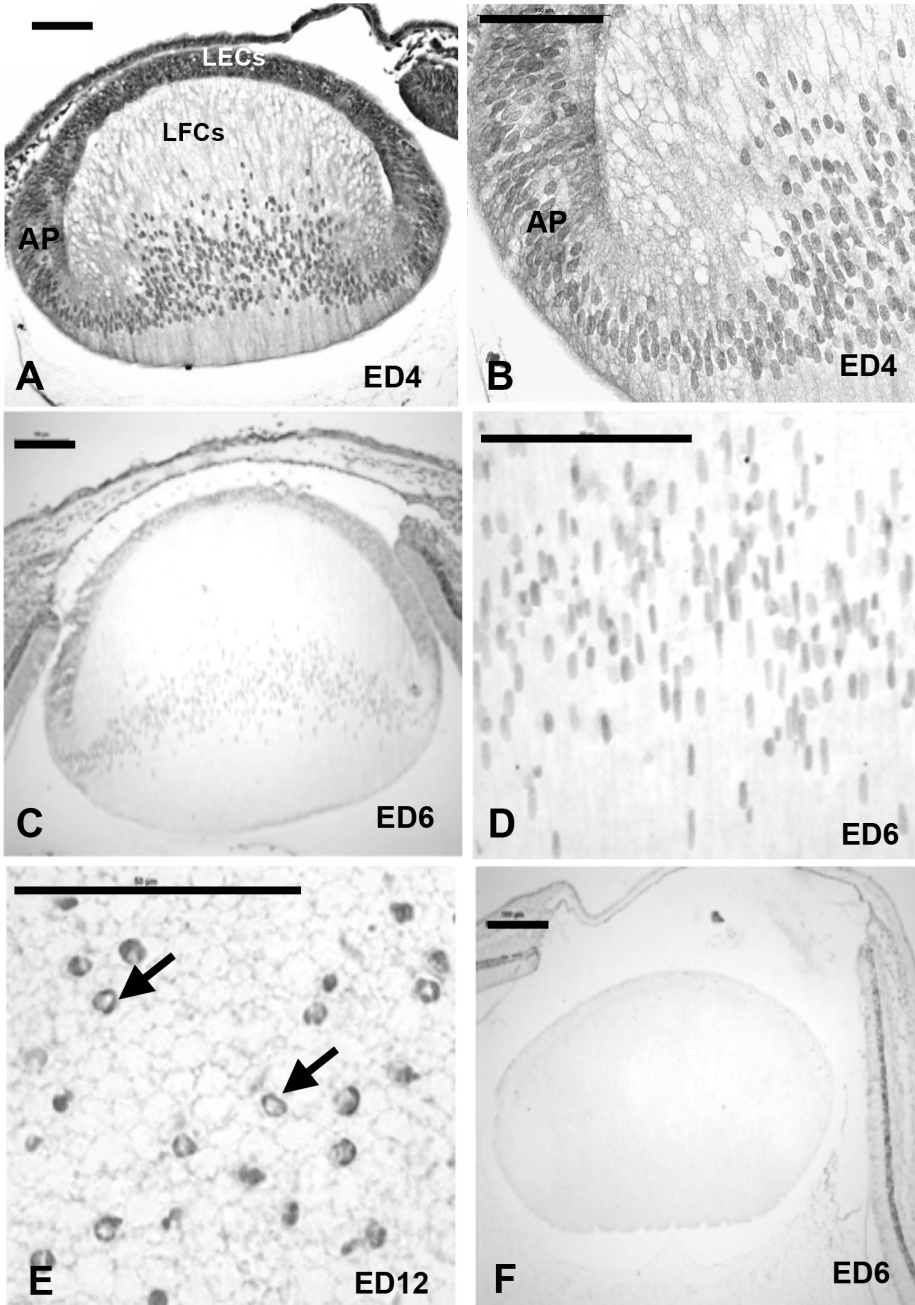
Spatiotemporal immunolocalization of Survivin in the chick embryo lens. **A**: Immunohistochemistry showed strong expression of Survivin at ED4 in the lens epithelial cells (LECs) and in the lens fiber cells (LFCs). Survivin staining was associated with the cytoplasm and nuclei in the LECs, but became mainly localized to the nuclei in the LFCs after passing through the transition zone at the annular pad (AP). **B**: Higher magnification revealed homogenous Survivin staining in LFC nuclei at ED4. **C**: In the ED6 lens, Survivin staining was associated with the LECs and the LFC nuclei. **D**: Higher magnification revealed homogenous Survivin staining in LFC nuclei at ED6. **E**: At ED12, Survivin staining was associated with marginalized chromatin of pyknotic nuclei of lens fiber cells undergoing the early stages of denucleation (arrows). **E**: ED6 negative control lens incubated with rabbit immunoglobulins instead of primary antibody demonstrates the specificity of Survivin immunostaining. Magnification bars are 100 μM except in **E** (50 μM).

We also examined the spatiotemporal pattern of Survivin expression in the developing chick lens using immunocytochemistry (Figure 2). Survivin staining was present in two pools in lens epithelial cells and fiber cells: cytoplasmic and nuclear. There was strong expression of cytoplasmic and nuclear Survivin in central and peripheral LECs at ED4, in the annular pad region and in the lens fiber compartment, specifically in the lens fiber cell nuclei (Figure 2A-B). At ED6, the staining followed a similar pattern to that observed at ED4 in the epithelial and fiber cell compartments (Figure 2C,D).

At ED12, the pattern of Survivin expression in the epithelium was similar to ED10 (data not shown). However, intensely stained pyknotic nuclei with marginalized chromatin were observed in the central lens fibers (Figure 2E). As negative control, the sections were incubated with rabbit immunoglobulin (Figure 2F).

The overall reduction in Survivin expression observed from E12 onwards in the developing chick lens was reflected in PCNA immunofluorescence staining used as an indicator of cell proliferation (Appendix 1). Overall, as would be expected, cell proliferation rates as determined with % PCNA labeled cells in different lens compartments diminished as development proceeded.

### Survivin gene and protein and proliferating cell nuclear antigen protein expression in chick lens epithelial cell primary cultures

**Figure 3:**
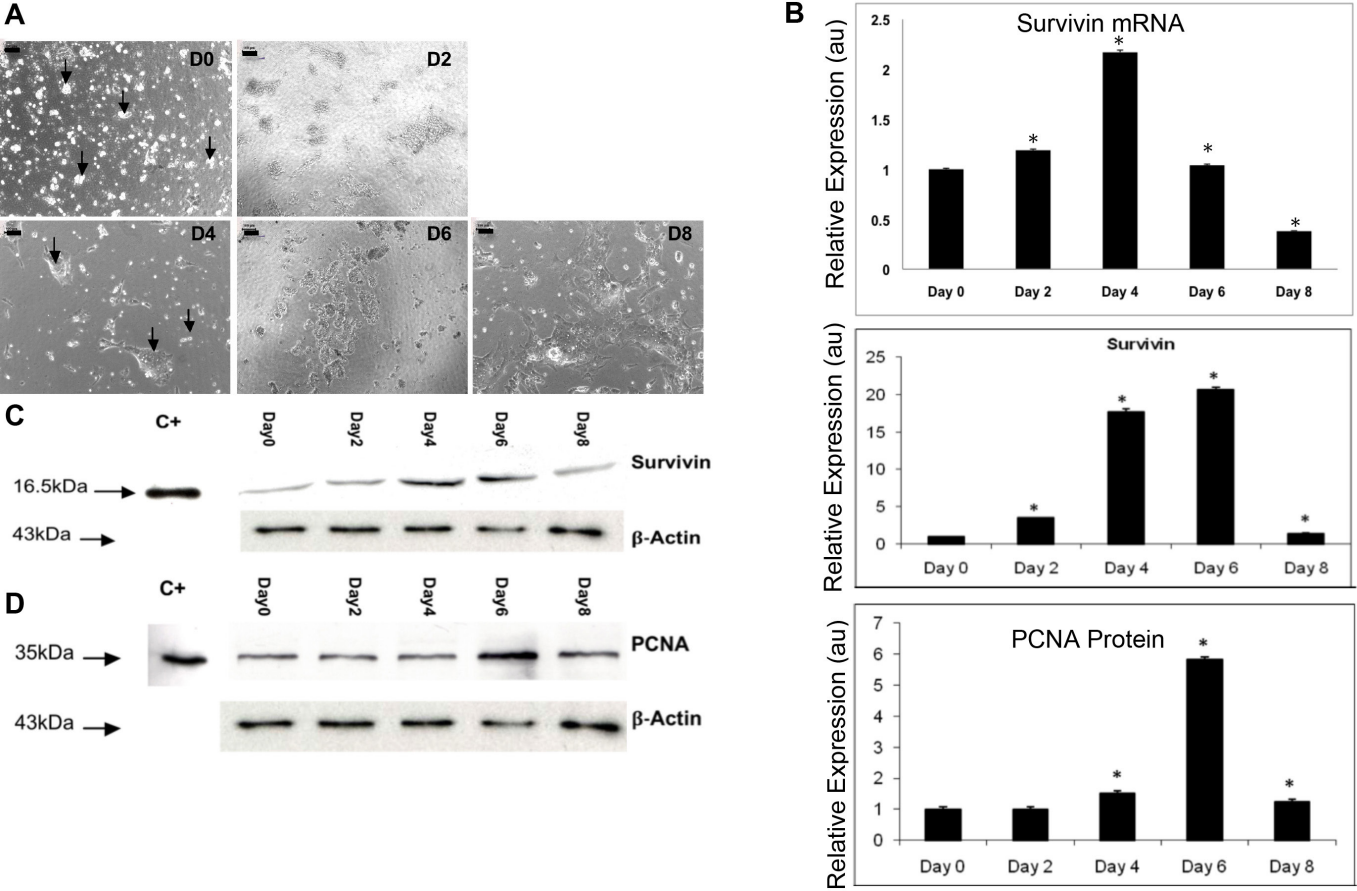
Survivin gene and protein and proliferating cell nuclear antigen (PCNA) protein expression in chick lens epithelial cell primary cultures. **A**: Culture of lens dissociated lens epithelial primary cell cultures revealed characteristic morphological changes using phase contract microscopy as cells progressively differentiated into lens fiber-like cells lentoids (arrows) between day 0 (D0) and day 8 (D8) of culture. Magnification bars=100 μM. **B**: Using QPCR, Survivin mRNA expression peaked at D0 and diminished thereafter. Statistical analysis of Survivin expression using QPCR revealed significant differences in expression levels between all stages studied and the calibrator D0 (*=p<0.05; n=3). **C**: Survivin expression peaked at D6 and diminished thereafter; thus, the peak of Survivin protein expression followed the peak of Survivin RNA expression. The amount of Survivin at each stage of culture was quantified with densitometry using the scan program and normalized regarding β-actin protein (*=p<0.05; n=3). **D**: PCNA expression peaked at D6. The amount of PCNA was quantified by densitometry using the scan program and normalized with respect to the β-actin protein (*=p<0.05; n=3). Chick embryo brain was used as a positive control C+) for Survivin and PCNA WBs. Representative western blots are shown for Survivin and PCNA. au=arbitrary units.

To further assess the expression of Survivin and PCNA during lens epithelial cell differentiation, we took advantage of the chick lens epithelial cell culture assay [29-31]. A progression of morphological development was observed as lens fiber-like cell lentoid development occurred (Figure 3A). Survivin expression in chick LECs was examined using QPCR (Figure 3B). Survivin mRNA expression peaked at day 4, diminishing thereafter. WB detected Survivin at 16.5 kDa (Figure 3C). Survivin protein expression peaked at day 6 of culture (two days after the peak of mRNA expression) and was dramatically downregulated thereafter. To investigate cell proliferation, WB was performed for PCNA. The anti-PCNA antibody detected a 35 kDa band (Figure 3D). WB revealed that PCNA expression peaked at day 6, but diminished thereafter.

### TdT-mediated biotin-dUTP nick-end labeling analysis in chick lens epithelial cell primary cultures

**Figure 4:**
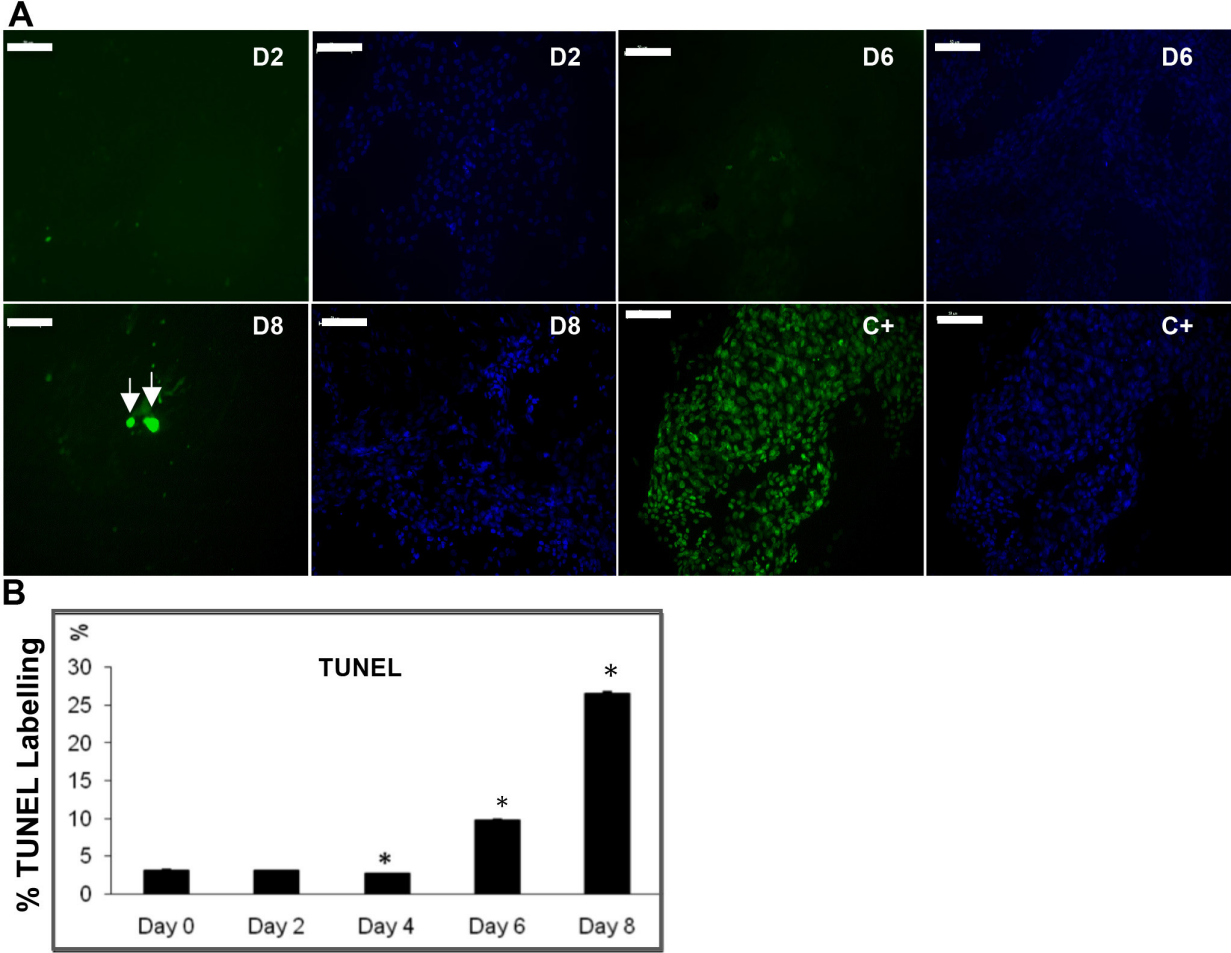
Terminal deoxynucleotidyl transferase (TdT)-mediated biotin-dUTP nick-end labeling (TUNEL) analysis in chick lens epithelial cell primary cultures. TUNEL staining to detect DNA fragmentation increased between day 0 (D0) and day 8 (D8) of culture. The histogram shows the mean and SEM of the % of TUNEL positive cells (arrows) at each stage. The % of TUNEL positive cells increased with time in culture, peaking at D8 and statistical significance was determined for each stage with respect to D0 (p<0.05; n=3). C+ indicates cultures predigested with DNase II before TUNEL labeling, as a positive control. Thus, the increase in TUNEL labeling observed through the culture period was inversely correlated with the profile of Survivin and PCNA expression in cultures.

TUNEL staining was used to examine denucleation in lens fiber-like cells (lentoids) in differentiating LECs in culture (Figure 4). TUNEL labeling remained low during the initial four days of cell culture, increased from day 6, and reached a peak at day 8. Thus, an inverse relationship between Survivin expression and TUNEL labeling, indicative of denucleation, was detected during the differentiation of LECs in vitro.

### Survivin and proliferating cell nuclear antigen expression in the early postnatal mouse lens

**Figure 5:**
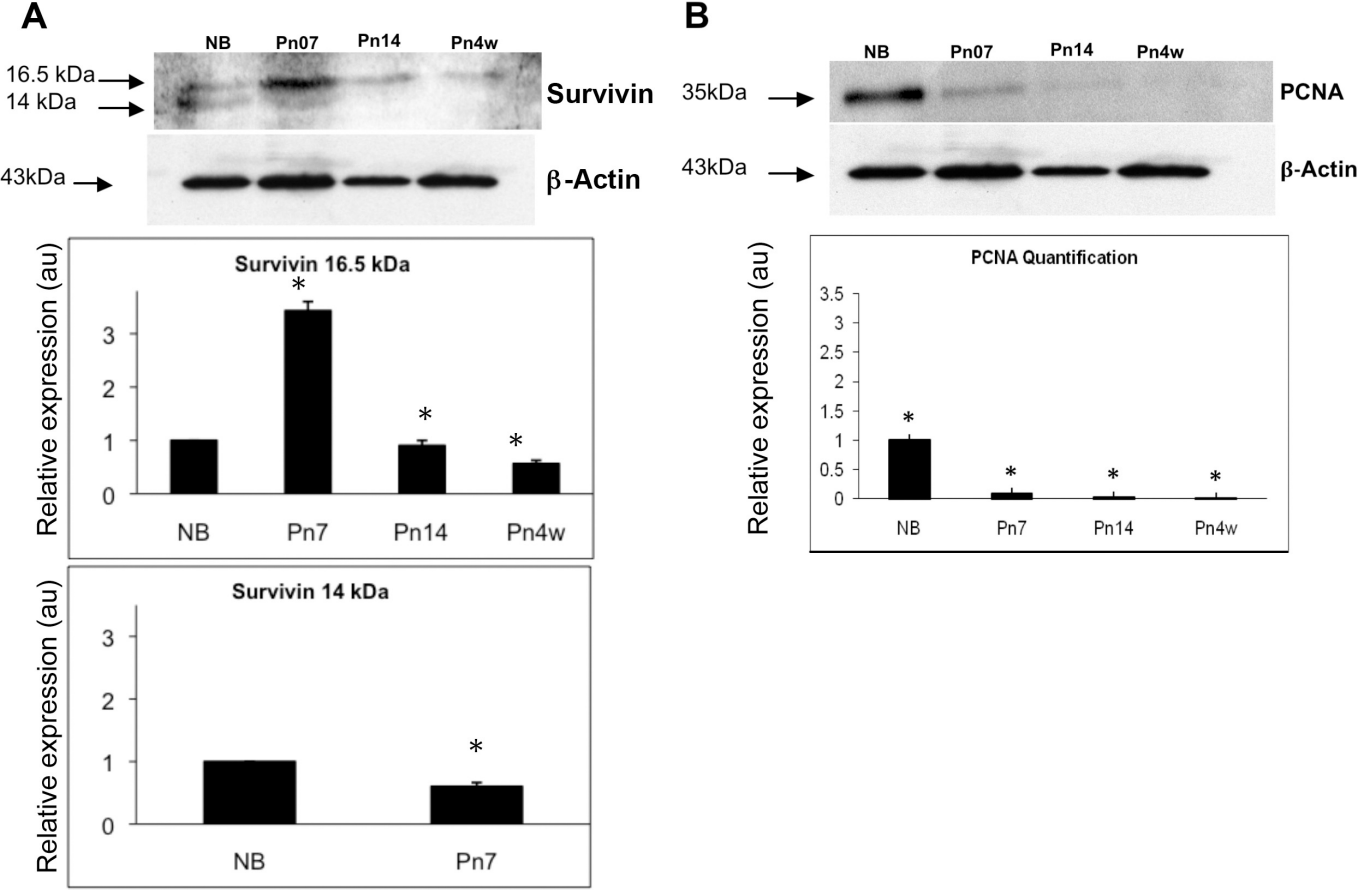
Survivin and proliferating cell nuclear antigen (PCNA) protein expression in the early postnatal mouse lens. **A**: During early stages of postnatal lens development, western blotting revealed two bands for Survivin at 16.5 kDa and 14 kDa. The 16.5 kDa band peaked at P7 and diminished in expression thereafter, while the 14 kDa band was expressed at lower levels at NB and P7 only. Relative expression levels of Survivin, determined with densitometry and normalized to β-actin protein, revealed statistically significant differences in levels of Survivin protein expression between the calibrator (NB) and the other stages studied (p<0.05; n=3). **B**: PCNA protein expression was downregulated between NB and 4 weeks. Relative expression levels of PCNA, determined with densitometry and normalized to β-actin protein using the scan program, revealed statistically significant differences in levels of PCNA protein expression between the calibrator NB and the other stages studied (p<0.05; n=3). Representative western blots are shown. au=arbitrary units.

To determine whether the patterns of expression of Survivin and PCNA detected during lens development in the chick lens were conserved between species, we also analyzed the postnatal mouse lens (Figure 5). Specific developmental stages of the mouse lens were chosen to correlate with the processes of lens development occurring in the chick lens. For example, the timing for the initiation of lens fiber cell differentiation in the chick lens is at ED2.5-ED3 (HH stages 17–18) [24], compared to E12.5 in the mouse lens [33], and the presence of a mature organelle-free zone at ED16 in the chick compared to P7 in the mouse. Differential expression of Survivin protein was found at all stages of postnatal lens development studied (NB, P7, P14, and at 4 weeks; Figure 5A). The Survivin 16.5 kDa wild-type band expression peaked at P7 and diminished thereafter. In addition to the 16.5 kDa band, corresponding to wild-type Survivin, a minor band was observed at 14 kDa at NB and P7, which was absent at P14 and 4 weeks. Cell proliferation was analyzed using WB for PCNA expression during postnatal mouse lens development. WB revealed a 35 kDa band corresponding to PCNA expression (Figure 5B). The band detected at the NB stage was the strongest observed in all samples. After NB, the intensity of the bands was steadily reduced through the P7 and P14 stages of development until 4 weeks, at which stage no band was observed. These data indicating a reduction in PCNA expression during mouse lens development were confirmed using PCNA immunofluorescence labeling (Appendix 2).

## Discussion

The differentiation of LECs into fiber cells is characterized by organelle loss, including denucleation [26,34], and involves many components of the cell death apparatus, such as caspase-3 and −6 [21,30,35-37]. However, organelle breakdown is not inhibited in mice lacking caspase-3, −6, −7, or a combination of caspase-3 and −6 [38]. It is therefore highly likely that organelle breakdown may occur through multiple pathways or functionally redundant networks [26]. For example, there is evidence that calpains [39] and the ubiquitin proteasome pathway [38,40,41] have roles to play in this process. Survivin could also be a factor involved in regulating organelle loss and denucleation during fiber cell differentiation.

The high conservation of the mechanisms regulating lens development between vertebrates species [42] offers a unique opportunity to use these different models to shed light on the role of Survivin during lens development. The results presented here show Survivin expression (mRNA and protein) during embryonic chick and postnatal mouse lens development and in a chick lens epithelial cell culture assay. Survivin expression in the lens was positively correlated with cell proliferation (PCNA labeling) and inversely correlated with lens fiber-like cells lentoid denucleation (TUNEL labeling).

### Expression pattern of Survivin, cell proliferation, and denucleation in the developing lens

Our results show that Survivin is expressed during embryonic chick and postnatal mouse lens development, with the highest expression during early stages of development and with a gradual reduction in levels of expression as development proceeded. Survivin was expressed in cytoplasmic and nuclear compartments in LECs, but became localized to the nuclei of the LFCs. The high expression of Survivin observed during the early stages of chick lens development is associated with the high rates of proliferation we observed using PCNA staining. Downregulation of Survivin coincided with reduced proliferation in LECs both in vivo and in vitro, as well as increased DNA fragmentation in lentoids in vitro. The correlation between Survivin expression and cell proliferation suggests a role for Survivin in cell cycle regulation during these early stages of lens development. These results support previous studies indicating that the expression of Survivin is associated with proliferation, as reported in fetal tissues [15,18] and cancer studies [43,44].

The expression of Survivin in LFCs suggests a role in fiber cell maturation. The requirement of Survivin for cell maturation has been described in erythroid cells, which like lens fiber cells lose their nuclei during differentiation [45,46]. In postnatal mouse lens development, Survivin was also developmentally regulated with highest expression at early stages of postnatal development. Cell proliferation in the postnatal mouse lens decayed abruptly after NB. Survivin expression increased significantly at P7 and then was progressively reduced in the lens, although still observed in all stages studied, suggesting an additional role for Survivin in the absence of proliferation. Interestingly, the protein analysis showed the presence of an additional band, albeit expressed at low levels, at 14 kDa at NB and P7. A similar band was previously observed in protein lysates from normal 12.5-day murine embryos [14], which identified this band with the Survivin splice variant 121 (Survivin121). According to these authors, it is unlikely that Survivin121 is involved in regulating the terminal caspases 3 and 7. Thus, different Survivin splice variants may have quite different biologic activities, and differential expression of such Survivin isoforms may adjust the balance between cell proliferation and apoptosis and/or denucleation of lens fiber cells. More work is therefore required on the significance of possible Survivin alternative splicing in the lens during development.

### Localization of Survivin in developing lens cells

Our results show that Survivin is detected in two pools in lens cells: cytoplasmic and nuclear. The nuclear localization of Survivin in epithelial and fiber cell compartments during early lens development is consistent with previous findings in neurons and tumor cells [18,47]. The mechanisms that control the Survivin nuclear-cytoplasmic localization are not known, but suggest an active role for Survivin in regulating cell viability and cell division [48]. Strong expression of Survivin in the nuclei of LECs may represent a role in mitotic events, since knockout of Survivin by homologous recombination results in cell proliferation defects leading to embryonic lethality [17]. Moreover, it has been suggested that during apoptotic stress Survivin redistributes from the cytoplasm to the nucleus to act as a physiologic switch to commit the cell to apoptosis and this spatial and functional regulation abolishes Survivin’s protective effect toward the apoptotic executors and commits the cell to apoptosis [49]. Thus, although the roles of the different pools of Survivin are not clear, the cytoplasmic pool of Survivin may inhibit the apoptosis pathway [50]. This cytoplasmic pool includes Survivin complexed with centromeres, microtubules, and other components of the mitotic apparatus [51,52]. Moreover, it has been suggested that the Survivin cytoplasmic pool may interplay with the apoptotic machinery controlling cell survival, but not cell proliferation [53], and it is possible that the presence of Survivin in the nuclei of central LFCs at ED12 in the chick lens is an early indicator of commitment to LFC denucleation. Furthermore, at P7 in the mouse, Survivin expression is at its highest. This is a time of rapid lens organelle-free zone formation, so high Survivin expression in the lens at this stage, particularly in LFC nuclei, may be important in this process, though clearly such a suggestion requires further in-depth functional analysis.

### Conclusions

Taken together, our results reveal that during lens development changes in levels of Survivin expression accompany changes in cell proliferation and differentiation rates, although these are not the only processes likely to be influenced by Survivin. Our data suggest that overall there is a positive relationship between Survivin expression and lens cell proliferation and an inverse relationship with denucleation during the differentiation of LECs into LFCs. In particular, LFC differentiation is accompanied by translocation of Survivin protein to the nuclei of these cells, where it associated with marginalized chromatin indicative of the early stages of denucleation. These data therefore suggest that Survivin may play an important role in vertebrate lens development. Further studies involving manipulation of Survivin expression and function in vivo and in vitro are now required to clarify the specific functions of Survivin in lens development, in particular in regulating lens cell proliferation and lens fiber cell differentiation, for example, through growth factor signaling pathways, including transforming growth factors [54], fibroblast growth factor [55] and Wnt/beta-catenin [56]. Moreover, work from the Menko laboratory has demonstrated that Survivin acts as a molecular switch in the differentiation process of LECs [57] and that blocking expression of members of the IAP family (including Survivin) resulted in a switch to apoptosis rather than differentiation of LECs [58]. Thus, the roles of IAPs in general, and of Survivin in particular in regulating LEC differentiation, merit further detailed functional analysis.

## Appendix 1. Spatio-temporal localization of PCNA in the chick embryo lens using immunofluorescence.

PCNA was used as a measure of cell proliferation. **A.** At ED4 (i-i’) and ED6 (ii-ii’) all nuclei of the epithelium and central fiber cells were positive for PCNA. **B.** Quantification of the percentage of PCNA positive nuclei revealed a progressive reduction in PCNA labeling in the central epithelium, peripheral epithelium and central fiber cells as development proceeded. Error bars represent the standard error of the mean. Statistical significance was determined for all groups with respect to ED 4 (*=p<0.05). To access the data, click or select the words “Appendix 1.” This will initiate the download of a compressed (pdf) archive that contains the file.

## Appendix 2. Spatio-temporal localization of PCNA during postnatal mouse lens development using immunofluorescence. A.

PCNA-positive nuclei were abundant at the NB stage, primarily in the lens epithelium (i-ii’). Staining was particularly intense in the peripheral lens epithelium (i, iii; arrowheads). Staining was also present, albeit faintly, in the lens fiber cells (ii and iii, asterisks). **B.** The percentage of PCNA-positive cells diminished in all lens compartments as development proceeded. Statistical significance was determined for all stages with respect to NB (*=p<0.05; n=3). Error bars represent SEM. LEC: lens epithelial cells. LFCs: lens fiber cells. To access the data, click or select the words “Appendix 2.” This will initiate the download of a compressed (pdf) archive that contains the file.
